# Enhancing natural killer cell anti-tumour activity through macrophage manipulation

**DOI:** 10.3389/fimmu.2025.1656925

**Published:** 2025-08-29

**Authors:** Natasha Palmer, Salim Khakoo, Tilman Sanchez-Elsner, Andres F. Vallejo

**Affiliations:** School of Clinical and Experimental Sciences, Faculty of Medicine, University of Southampton, Southampton, United Kingdom

**Keywords:** macrophages, natural killer cells, immune crosstalk, cancer, immunotherapy

## Abstract

The tumour microenvironment (TME) is a complex and dynamic environment containing diverse cellular, stromal and soluble factors, that collectively influence cancer progression, immune evasion and therapeutic resistance. Among the immune components of the TME, macrophages and natural killer (NK) cells are key players, whose interactions, particularly their crosstalk, critically shape anti-tumour immunity. The macrophage–NK cell interplay can either promote or suppress immune responses depending on the context, representing both a challenge and a therapeutic opportunity. NK cells are key effectors capable of recognising and eliminating malignant cells without prior sensitisation, whereas macrophages exhibit remarkable plasticity, functioning as either promoters or suppressors of tumour immunity depending on their activation state. This review focuses on current strategies to harness macrophages in cancer therapy, including phenotype repolarisation, selective depletion, and disruption or enhancement of the macrophage-NK cell crosstalk to enhance NK cell-mediated tumour surveillance. Finally, we highlight emerging technologies, such as single-cell RNA sequencing, spatial transcriptomics, and proteomics, as powerful tools to elucidate the dynamic interplay between macrophages and NK cells and inform the next generation of immunotherapeutic interventions.

## 1 Macrophages: versatile regulators of tissue homeostasis and immunity

Macrophages are innate immune cells with essential roles across homeostasis, inflammation, and cancer. They originate from multiple developmental pathways, including embryonic progenitors such as yolk sac and foetal liver precursors, which give rise to long-lived tissue-resident populations like microglia in the brain, Kupffer cells in the liver, and alveolar macrophages in the lung (1, 2). These populations are maintained independently of circulating monocytes throughout adult life (3). By contrast, macrophages associated with inflammation or pathology are typically derived from adult haematopoietic stem cells via monocytes, which are recruited to tissues in response to chemotactic signals and differentiate in situ (4–6).

Across tissues, macrophages carry out a core set of functions, including the phagocytosis of pathogens, apoptotic cells, and debris; the presentation of antigens via Major Histocompatibility Complex (MHC) molecules to T cells; and the secretion of cytokines and chemokines that modulate both innate and adaptive immune responses (7). However, their phenotype and function are shaped by the local tissue microenvironment and inflammatory cues, giving rise to considerable heterogeneity.

*In vitro* models have classically categorised macrophage polarisation into ‘M1’ and ‘M2’ phenotypic states, pro-inflammatory and anti-inflammatory respectively, based on stimulation with microbial products (e.g., LPS, IFN-γ) (8) or anti-inflammatory cytokines (e.g., IL-4, IL-13) (9). While this M1/M2 paradigm has provided a useful conceptual framework, it does not adequately reflect the complexity of heterogeneity of macrophage phenotypes observed *in vivo*, particularly within pathological settings such as the tumour microenvironment (TME). Tumour-associated macrophages (TAMs), for instance, do not conform neatly to M1 or M2 phenotypes but rather exhibit a spectrum of activation states that can simultaneously support immunosuppression, tissue remodelling, and tumour progression (10, 11). Unravelling this functional and phenotypic diversity remains a major focus in immunology, with implications for both fundamental biology and therapeutic targeting.

### 1.1 Activating and inhibitory receptors: regulating macrophage activation

Macrophage activation is regulated by a balance of stimulatory and inhibitory signals that enable these plastic cells to dynamically adapt to their environment. Among the main activating pathways are pattern recognition receptors (PRRs), notably Toll-like receptors (TLRs). TLRs recognise conserved pathogen-associated molecular patterns (PAMPs) and danger-associated molecular patterns (DAMPs), initiating downstream signalling cascades such as NF-κB activation. This leads to the production of pro-inflammatory cytokines, including TNF-α, IL-6 and IL-12 (12, 13). Co-stimulatory receptors like CD40 also promote macrophage activation, particularly through interaction with CD40L on CD4^+^ T cells, resulting in similar cytokine production (14–17). Triggering Receptor Expressed on Myeloid Cells-1 (TREM-1) further amplifies inflammatory responses by synergising with TLR signalling (18), whereas TREM-2 is associated with a regulatory phenotype, supporting phagocytosis and tissue remodelling (19).

Conversely, macrophage activation is tightly controlled by inhibitory receptors that prevent excessive tissue damage and maintain immune homeostasis. The checkpoint receptor programmed death-1 (PD-1) and its ligand PD-L1 modulate macrophage function by dampening inflammatory responses (20). The scavenger receptor Macrophage Receptor with Collagenous Structure (MARCO), also plays a immunoregulatory role by mediating the clearance of apoptotic cells and microbial components (21, 22). Additional regulatory pathways include the CD47–SIRPα axis, which inhibits macrophage phagocytosis and is frequently exploited by cancer cells to avoid clearance (23).

## 2 Natural killer cells: key players in immune surveillance and tumour defence

Natural killer (NK) cells are critical effectors of the innate immune system, capable of detecting and eliminating transformed or infected cells. These cytotoxic lymphocytes develop in the bone marrow and undergo maturation in secondary lymphoid organs, such as the spleen, lymph nodes and tonsils (24). Once in circulation, where they comprise 5-15% of peripheral blood lymphocytes, NK cells traffic to diverse tissues, including lymphoid and non-lymphoid sites like the liver and lungs (25). NK cell populations are broadly divided into two functional subsets: the cytotoxic CD56^dim^ subset, which predominates in peripheral blood and the spleen, and the CD56^bright^ subset, enriched in secondary lymphoid tissues and characterised by robust cytokine production, including IFN-γ, TNF-α, and GM-CSF (26–28). CD56^bright^ NK cells also secrete chemokines such as CCL3–5 and CXCL8 (24, 29), contributing to immune cell recruitment and orchestration of early immune responses.

### 1.2 Activating and inhibitory receptors: coordinating NK cell responses

NK cell function is intricately regulated by a balance of activating and inhibitory germline-encoded receptors. Activating receptors, including NKG2D, DNAM-1, and natural cytotoxicity receptors (NCRs), recognise ligands that are upregulated on stressed, infected, or malignant cells (30–32). Conversely, inhibitory receptors, such as those within the killer immunoglobulin-like receptor (KIR) family and the NKG2A–CD94 heterodimer, monitor the expression of self-MHC class I molecules, preventing autoreactivity (33–35). This delicate receptor balance ensures that NK cells maintain tolerance to healthy cells while retaining the ability to target cells that have downregulated MHC class I expression, a common immune evasion mechanism in tumour cells. Thus, NK cells provide a crucial layer of immune surveillance that operates independently of T cell recognition (36).

The activation of NK cells typically requires the engagement of multiple activating receptors; however, CD16 (FcγRIIIa), the receptor responsible for mediating antibody-dependent cellular cytotoxicity (ADCC), can induce cytotoxic responses in the absence of other activating signals (37, 38). The KIR family, which includes both activating and inhibitory isoforms, plays a critical role in NK cell education, ensuring functional licensing and the development of tolerance to self (39). This process enables NK cells to discern between healthy and aberrant cells, with the clonal distribution of activating and inhibitory receptors across the NK cell repertoire allowing for context-dependent responses to both tumorigenic and infectious cells (40).

## 3 The role of macrophage-NK cell communication in immune defence

Macrophages and NK cells influence each other through dynamic, bidirectional interactions that shape immune responses (**Table 1**), and often these interactions are dependent on their anatomical context. Macrophages are largely tissue-resident and adapt to the specific microenvironment of their organ (49). While NK cells are traditionally described as circulating between blood and lymphoid tissues, they can be recruited to sites of inflammation or tumours (50–52), and subsets of tissue-resident NK cells have been identified in organs such as the liver and uterus during pregnancy, where they exhibit distinct phenotypic and functional properties (53, 54). In peripheral tissues, including the liver and lung, macrophages can activate NK cells through direct cell-to-cell contact and the secretion of soluble mediators. Notably, co-culture studies have demonstrated that blocking activating receptors such as DNAM-1 or 2B4, or neutralising IL-18, leads to reduced NK cell-derived IFN-γ (41), underscoring the importance of both receptor–ligand interactions and cytokine signalling in this axis. In addition, macrophage-derived IL-1β and type I interferons, particularly IFN-β, have been shown to upregulate the expression of activating NK cell receptors including NKp44 and NKG2D, thereby enhancing IFN-γ production (43).

**Table 1:** Key interactions between macrophages and NK cells.

Interaction	Mediators	Cellular response	Physiological significance	References
Macrophage modulation of NK cells: direct cell-to-cell contact	NKG2D-MICA/MICB, DNAM-1-CD112/CD155, 2B4-CD48, CD40-CD154	NK cell activation, IFN-γ production	Enhanced immune response	(41, 42)
Macrophage modulation of NK cells: soluble mediators	IL-15, IL-18, IL-1β, IL-23, IFN-γ, IFN-β	NK cell priming, enhanced cytotoxicity, cytokine production	Enhanced immune response	(41, 43)
NK cell modulation of macrophages: direct cell-to-cell contact	CD40-CD154	Enhanced macrophage phagocytosis	Improved pathogen clearance	(44)
NK cell modulation of macrophages: soluble mediators	IFN-γ	Enhanced macrophage phagocytosis	Improved pathogen clearance	(45, 46)
NK cell killing of macrophages	NKG2D ligands	Elimination of overactivated macrophages	Regulation of inflammation	(47)
Macrophage self-protection	HLA-E	Macrophage resistance to NK cell lysis	Maintenance of macrophage population	(48)

The table summarizes key modes of macrophage-NK cell crosstalk, including direct cell-to-cell contact and soluble mediators, the specific molecular mediators involved, resulting cellular responses, and their physiological significance. These interactions highlight the bidirectional modulation between macrophages and NK cells, contributing to immune activation, pathogen clearance, and regulation of inflammation.

The functional role of NKG2D-mediated signalling has been particularly well characterised in uterine NK cells, where recognition of macrophage-expressed MICA drives robust IFN-γ responses (42). Conversely, NK cells can reciprocally activate macrophages. Engagement of CD40 on macrophages by CD154 expressed on NK cells induces the production of pro-inflammatory cytokines (55, 56), and macrophages from CD40-deficient mice exhibit impaired phagocytic activity in the presence of NK cells (44). Additionally, NK cell-derived IFN-γ can reprogramme immunosuppressive macrophages towards a more immunostimulatory phenotype (45), characterised by enhanced secretion of IL-12, TNF-α, and CXCL chemokines (57). This reciprocal activation is further amplified by a positive cytokine feedback loop: macrophage-derived IL-12, IL-15, and IL-18 activates NK cells, which in turn produce IFN-γ, TNF-α, and GM-CSF that further stimulate macrophage function and inflammatory cytokine production (46, 58, 59).

Importantly, this cooperative relationship is tempered by regulatory mechanisms that prevent excessive inflammation and preserve tissue integrity. For instance, type I interferons and Toll-like receptor (TLR) agonists can upregulate NKG2D ligands (such as MICA and ULBP1–3) on macrophages (42, 47), promoting their recognition and lysis by NK cells via NKG2D (47). However, inhibitory signalling through NKG2A, which recognises HLA-E on macrophages, restrains NK cell-mediated cytotoxicity (48). Macrophages activated by inflammatory stimuli express higher levels of HLA-E, providing increased protection against NK cell killing (48). Additionally, blockade of activating NK cell receptors, including NKp46 and DNAM-1, reduces macrophage susceptibility to NK cell-mediated cytotoxicity (60).

Together, these observations emphasise a finely tuned balance between NK cell-mediated activation and restraint, ensuring sufficient immune surveillance without depleting critical macrophage populations. In the context of cancer, selectively modulating this axis, such as through temporal regulation of NKG2D ligand expression, may offer a strategy to eliminate immunosuppressive tumour-associated macrophages while preserving homeostatic ones in healthy tissues.

## 4 Macrophage-NK cell crosstalk in the tumour microenvironment: balancing immunity and tolerance

**Figure 1:**
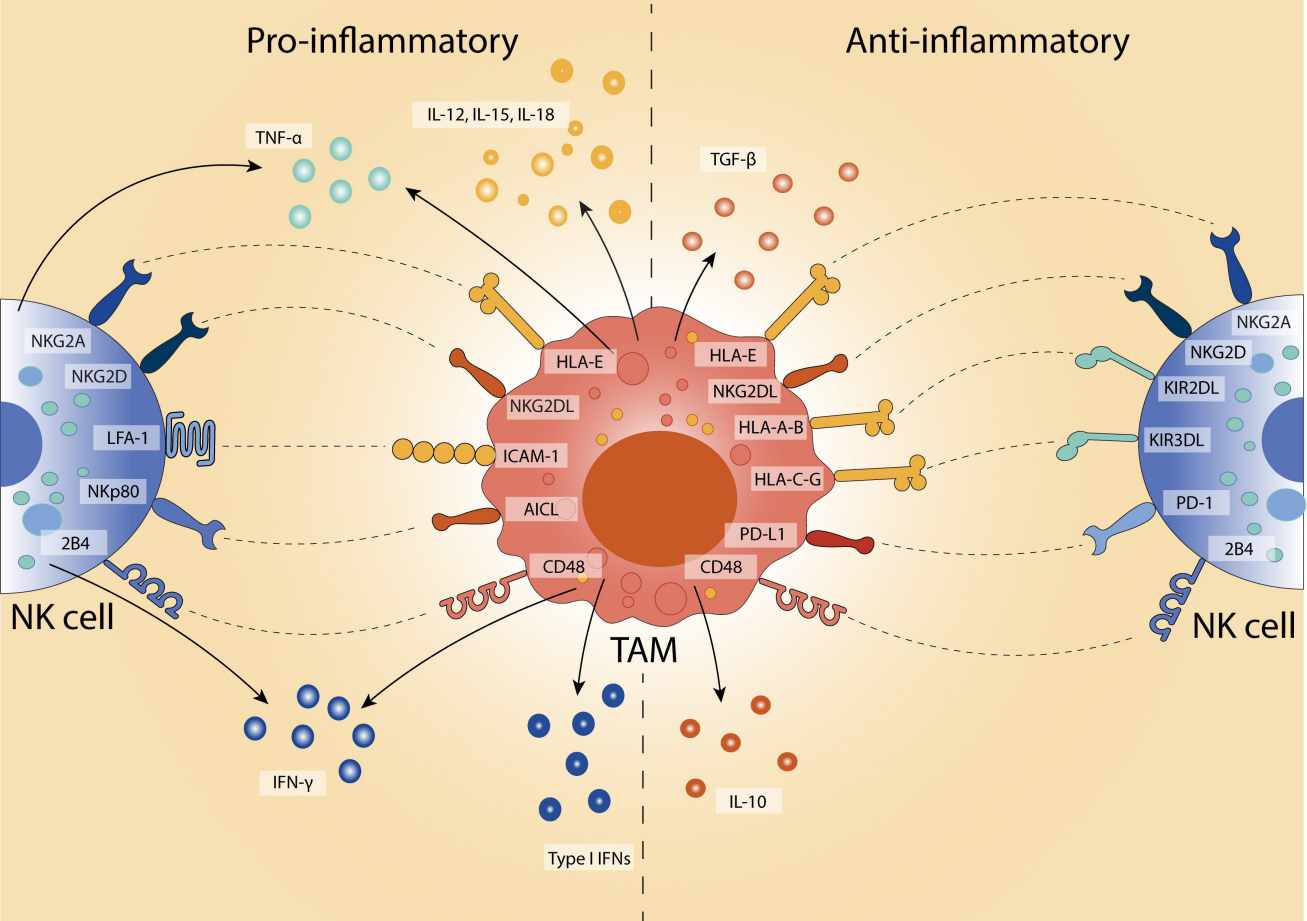
Polarised TAM-NK cell interactions in the TME. TAMs modulate NK cell activity depending on their polarisation state. Pro-inflammatory TAMs (left) promote anti-tumour responses by secreting IL-12, IL-15, IL-18, and TNF-α, and enhancing NK cell activation and IFN-γ production through engagement of activating receptors (e.g., NKG2D, NKp80, LFA-1). In contrast, anti-inflammatory TAMs (right) facilitate immune suppression via IL-10 and TGF-β, and upregulating inhibitory ligands (e.g., PD-L1, HLA-E, NKG2DL) that engage inhibitory NK cell receptors (e.g., PD-1, NKG2A, KIRs). This bidirectional crosstalk shapes the tumour microenvironment by either enhancing or suppressing NK cell cytotoxicity.

TAMs and NK cells are key components of the TME, each contributing in distinct and often opposing ways, with their dynamic crosstalk shaping the TME (**Figure 1**). TAMs are typically the most abundant immune population within the TME, comprising up to 50% of infiltrating immune cells and contributing significantly to tumour structure and immunoregulation (61, 62). While they share some functional overlap with myeloid-derived suppressor cells (MDSCs), such as the ability to dampen anti-tumour immunity, TAMs are generally more differentiated and tissue-resident, whereas MDSCs represent a heterogeneous population of immature myeloid cells that expand during cancer and other chronic inflammatory conditions (63).

In early-stage tumours, macrophages may exhibit immunostimulatory phenotypes and exert tumoricidal functions (64), partly through nitric oxide production (65), and have been associated with improved clinical outcomes in colorectal (66), lung (67), ovarian (68), and breast cancers (69). However, as tumours progress, the local cytokine milieu shifts to favour anti-inflammatory cues, such as IL-10 and CSF1, driving the recruitment of monocytes and their subsequent differentiation into immunosuppressive TAMs (68, 70). These TAMs often adopt a phenotype resembling anti-inflammatory macrophages and are associated with poor prognosis across a wide spectrum of malignancies, including pancreatic, breast, endometrial, and brain cancers, as well as lymphomas and melanomas (7).

In contrast, NK cells are potent effectors of anti-tumour immunity. Their ability to directly lyse malignant cells and secrete pro-inflammatory cytokines such as IFN-γ renders them crucial for early tumour surveillance. Preclinical models demonstrate that prolonged NK cell depletion accelerates tumour progression (71–73), while clinical studies link reduced NK cell infiltration or impaired function with increased metastatic potential and recurrence, particularly in colorectal, head and neck, and pharyngeal cancers (74–77). Despite robust *in vitro* cytotoxicity against tumour targets such as melanoma, adoptive NK cell transfer has yielded limited clinical efficacy, often due to suppressive factors within the TME, including downregulation of activating receptors such as NKG2D (78).

### 4.1 Soluble mediators of communication: cytokine-driven modulation

Macrophage-NK cell communication within the TME is shaped by both soluble mediators and direct cell-cell interactions. Although these cells often reside in close proximity in the TME, cytokines such as TAM- and tumour-derived IL-10 still exert broad immunosuppressive effects, dampening both macrophage and NK cell effector functions (46, 79). Interestingly, *in vitro* addition of IL-15 can restore NK cell cytotoxicity in the presence of IL-10, suggesting that IL-10’s suppressive effects may be context-dependent (80). TGF-β, a prominent immunoregulatory cytokine secreted by both TAMs and tumour cells, suppresses NK cell activation by downregulating NKG2D and NKp30 (81), while concurrently promoting macrophage polarisation toward an immunosuppressive phenotype and reducing pro-inflammatory cytokine output (82). In gastric cancer, macrophage-derived TGF-β induces a marked functional impairment in NK cells, characterised by reduced IFN-γ production and diminished expression of NKp30, NKp46, and 2B4 (83). Notably, this suppression can be partially reversed by exogenous IL-15 (84).

NK cells also exert feedback effects on macrophages. In prostate cancer, NK cell-derived IL-8 recruits macrophages and skews their polarisation toward a tumour-promoting phenotype (85). Similarly, activated NK cells release cytotoxic granules containing effector molecules such as granzymes which can act on macrophages. For example, granzyme A can stimulate macrophages to produce pro-inflammatory cytokines including IL-6, IL-8, IL-1β and TNF-α (86–88). Conversely, macrophages, particularly those with anti-tumour activity, are a major source of type I interferons (IFN-α and IFN-β), which are essential for NK cell development and activation (89). These interferons reduce TAM frequency, promote inflammatory polarisation, and induce chemokines such as CXCL10 and CXCL11, which recruit NK cells via CXCR3 (90–94). IFN-β also enhances NK cell cytotoxic potential by upregulating NKG2D and inducing IL-15, a cytokine critical for both macrophage activation and NK cell survival, proliferation, and function (43). TAMs within the TME frequently produce IL-12, IL-15, and IL-18, a group of cytokines that work together to sustain NK cell activity (44, 48, 95, 96).

TNF-α, produced by both macrophages and NK cells, reinforces this axis by suppressing anti-inflammation-associated gene expression and enhancing IL-15 signalling pathways in NK cells (97, 98). Via TNFR1 and TNFR2, TNF-α activates pro-inflammatory signalling cascades that can culminate in tumour cell death (99, 100). Importantly, the outcome of macrophage–NK cell crosstalk depends on the prevailing balance of stimulatory versus inhibitory cytokines and on the spatial and temporal context of their interaction. Therapeutic strategies must therefore account for the heterogeneity of cytokine networks and macrophage polarisation states within individual tumours.

### 4.2 Receptor-ligand interactions: immune synapses in tumour immunity

In addition to soluble factors, direct contact between macrophages and NK cells is also important in shaping immune responses. NK cell activity is modulated through a network of activating and inhibitory receptors that respond to ligand expression on both tumour cells and myeloid populations. For instance, NKp46-deficient mice exhibit impaired tumour control in lymphoma and melanoma models (101–104), while reduced NKp30 and NKp46 expression in patients with leukaemia or cervical cancer correlates with poorer outcomes (105–107). Conversely, high expression of DNAM-1 enhances NK cell responses against both haematological malignancies and solid tumours (108).

Within the TME, tumour-derived CSF1 induces macrophage expression of NKG2D ligands, including MICA/B and ULBPs, thereby promoting NK cell activation (109). However, chronic NKG2D stimulation, as observed in acute myeloid leukaemia and hepatocellular carcinoma (HCC), can lead to NK cell exhaustion (110–112). Similarly, the 2B4-CD48 axis supports NK cell activation and IFN-γ production (41, 47, 113), but persistent engagement in the context of HCC drives functional impairment (114). Other macrophage-expressed ligands, such as AICL and ICAM-1, engage NKp80 and LFA-1 on NK cells, respectively, facilitating activation and migration (115–117). Notably, dectin-1, a C-type lectin receptor expressed by macrophages, recognises tumour-specific glycan structures and initiates signalling pathways that enhance NK cell cytotoxicity (118).

These receptor-ligand interactions act as important checkpoints that help control anti-tumour immune responses. However, because these signals are highly dynamic and influenced by the suppressive nature of the TME, therapeutic strategies need to be carefully tailored. New technologies, such as co-culture systems, single-cell transcriptomics, and spatial profiling, will be key to mapping these interactions within the TME and uncovering precise targets for immune-based therapies.

## 5 Harnessing macrophages: innovative approaches to target cancer

While T cells have been the main target of cancer immunotherapy for years, focusing on TAMs offers distinct advantages. Unlike T cells, which face challenges such as exhaustion, antigen escape, and TME infiltration (119–122), macrophages are already abundant and well-established within tumours (61, 62). TAMs play a pivotal role in shaping the TME by promoting tumour growth, suppressing T cell function, supporting angiogenesis, and remodelling the extracellular matrix (123–125). Additionally, they are key regulators of other immune populations (126–129); within the TME, the immunosuppressive interplay between TAMs and NK cells can further dampen effective anti-tumour responses. As they are less reliant on tumour-specific antigens, therapies targeting TAMs may also be less susceptible to immune evasion and more broadly applicable (130, 131).

Previous reviews have discussed immunotherapeutic strategies targeting TAMs (46, 132), yet few studies have addressed the therapeutic potential of modulating TAMs specifically to augment NK cell function. This remains a significant and underexplored area, with emerging evidence suggesting that TAM modulation can shape NK cell recruitment, activation and cytotoxicity. A summary of therapeutic approaches under clinical and preclinical investigation is outlined in **Table 2**. Current therapeutic strategies focus on three main approaches: reprogramming macrophage phenotype, depleting immunosuppressive subsets, and modulating TAM–NK cell interactions to restore cytotoxic activity.

**Table 2:** Cancer therapeutic strategies targeting TAMs to enhance NK cell effector function.

Therapeutic strategy	Study type	Tumour context	Effect on TAM-NK crosstalk	References
TLR4 agonist (LPS)	Preclinical	Ovarian cancer	Induces pro-inflammatory TAMs: ↑ NK cell activation	(133)
TLR3 agonist (poly(I:C))	Preclinical	Lung	Induces pro-inflammatory TAMs and MICA expression: ↑ NK cell cytotoxicity	(134)
TLR2 agonist (β-glucan)	Preclinical	Melanoma	TAM activation: ↑ NKG2D expression	(135)
Anti-MARCO antibody	Preclinical	Melanoma, breast cancer, solid tumours	Reprogrammes TAMs: ↑ IL-15 secretion and NK cell cytotoxicity	(136–139)
Anti–Clever-1 antibody (FP-1305 and Bexmarilimab)	Early-phase clinical	Advanced solid tumours	Reprogrammes TAMs: ↑ NK cell numbers and IFN-γ production	(140, 141)
Checkpoint inhibitors (PD-1/PD-L1)	Clinical	Osteosarcoma, ovarian cancer	Restores TAM phagocytosis: ↑ NK cell activation	(20, 142, 143)
Checkpoint inhibitors with anti-TREM2 or anti-MARCO antibodies	Preclinical	Ovarian cancer	TAM repolarisation: ↑ NK cell infiltration	(136, 144)
Genetic ablation of CSF1R	Preclinical	Breast cancer	↓ TAM accumulation: ↑ NK cell activation	(145)
Small molecule blockade of CSF1R (BLZ945)	Preclinical	Glioma	Reprogrammes TAMs and ↑ antigen presentation genes: ↓ NK cell infiltration	(146, 147)
CSF1R inhibitor (Emactuzumab)	Early-phase clinical	Diffuse-type tenosynovial giant cell tumours,	↓ TAM accumulation: ↑ NK cell activation (pre-clinical)	(148, 149)
CCL2 blockade/CCR2 antagonists	Preclinical	Hepatocellular carcinoma	↑ TAM accumulation: ↑ NK cell activation, cytotoxicity and IFN-γ production	(150–152)
CCR2 antagonist (PF-04136309)	Early-phase clinical	Pancreatic ductal adenocarcinoma	↓ TAM accumulation: ↑ NK cell activation (pre-clinical)	(153)
CAR-T cells targeting FRβ+ TAMs	Preclinical	Solid tumours	Selective depletion of TAMs: effect on NK cells not explored	(154)
TGF-β blockade	Preclinical	Gastric and breast cancer	Induces pro-inflammatory TAMs: ↑ NK cell activation	(83, 145, 155)
Axl or GAS6 blockade	Preclinical	Melanoma, breast and pancreatic cancer	Induces pro-inflammatory TAMs: ↑ NK cell activation	(156, 157)

The table displays preclinical and clinical approaches that modulate TAMs to improve NK cell activation, recruitment, or cytotoxicity. Strategies include antibody-based depletion or reprogramming of TAM subsets (e.g., anti-MARCO, anti–Clever-1), immune checkpoint blockade and inhibition of macrophage recruitment (e.g., CCL2/CCR2 axis). While most approaches remain preclinical, several agents are under early-phase clinical evaluation and show potential to restore NK cell function in the TME.

↑ represents increase and ↓ represents decrease.

### 5.1 Repolarising TAMs: shifting macrophages to anti-tumour action

**Figure 2:**
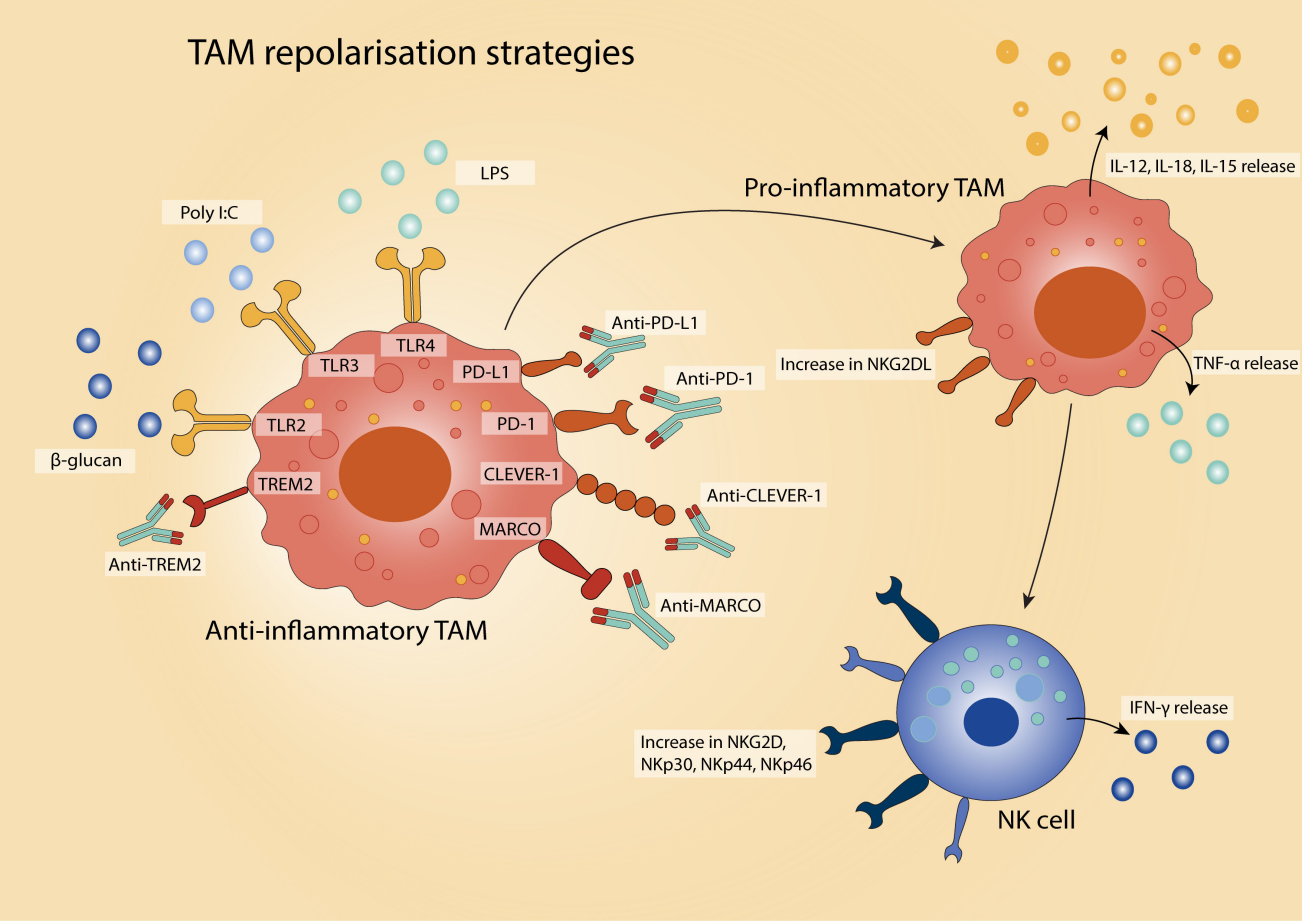
Strategies for TAM repolarisation in the TME. There are various approaches to reprogramme TAMs from an anti-inflammatory, pro-tumour phenotype to a pro-inflammatory, anti-tumour state. Therapeutic strategies include activation of pro-inflammatory-inducing pathways via agents targeting Toll-like receptors (TLRs), and immune checkpoint blockade (e.g., anti-PD-1/PD-L1) to relieve TAM-mediated immunosuppression. The reprogrammed TAMs exhibit enhanced phagocytic activity, antigen presentation, and pro-inflammatory cytokine secretion, contributing to improved anti-tumour immunity.

One strategy to increase the anti-tumour response involves reprogramming TAMs from a tumour-promoting state toward a more pro-inflammatory, tumour suppressive phenotype, thereby enhancing NK cell recruitment and effector function (**Figure 2**). *In vitro*, macrophages described as ‘M2-like’ can be repolarised to an ‘M1-like’ phenotype, promoting IFN-γ secretion and restoring cytotoxicity in resting NK cells against multiple tumour targets, achieving activity comparable to IL-12-conditioned NK cells (41, 48, 135). Repolarised macrophages also secrete increased levels of IL-12, IL-18, and TNF-α (41), and upregulate NK cell-activating receptors such as NKp30, NKp44, NKp46, and NKG2D (47, 135). Notably, these repolarised macrophages exhibit resistance to NK cell-mediated cytotoxicity (48), suggesting a cooperative rather than antagonistic relationship between the two cell types.

Toll-like receptor (TLR) agonists represent a widely studied class of agents capable of driving this phenotypic shift. For instance, stimulation with lipopolysaccharide (LPS, TLR4 agonist) enhances the ability of TAMs isolated from ovarian cancer to activate NK cells and promote tumour cell lysis (133). Similarly, poly(I:C) (a synthetic TLR3 ligand) induces pro-inflammatory phenotype polarisation of alveolar macrophages in lung cancer, resulting in heightened NK cell cytotoxicity and suppression of metastatic growth (134). In uterine macrophages, poly(I:C) also upregulates the NKG2D ligand MICA, enabling robust NK cell activation via NKG2D engagement (42). In melanoma, β-glucan (a TLR2 agonist) increases NKG2D expression on NK cells and enhances tumour control in a manner dependent on NK cell presence (135).

Beyond TLR-based interventions, antibody-mediated targeting of TAM-associated surface receptors offers an additional strategy to reprogramme macrophage function and restore NK cell responsiveness. In melanoma, TAMs expressing the scavenger receptor MARCO are found near NK cells (136, 137). Antibody blockade of MARCO reprogrammes TAMs toward an immunostimulatory phenotype, enhances IL-15 secretion, and increases NK cell infiltration and cytotoxicity (136, 138). In preclinical breast cancer models, anti-MARCO treatment also reduces tumour burden and metastatic dissemination (137). A similar effect has been observed with antibodies targeting Clever-1 (also known as stabilin-1), which boosts NK cell numbers and IFN-γ production in patients with advanced-stage solid tumours (140). Treatment with the anti–Clever-1 antibody bexmarilimab in patients with solid tumours has similarly been associated with increased IFN-γ and NK cell activation markers (141).

Immune checkpoint blockade has also emerged as a strategy to reprogramme TAMs. TAMs express both PD-1 and PD-L1, and blockade of this axis not only restores tumour cell phagocytosis but also enhances NK cell activation (20). Macrophage-targeted anti–PD-L1 therapy has been shown to increase IFN-γ production by NK cells (142), while anti–PD-1 treatment in osteosarcoma models expands the anti-tumour macrophage population (143). Importantly, combining immune checkpoint inhibitors with TAM-targeting agents such as anti-MARCO or anti-TREM2 antibodies enhances therapeutic efficacy in preclinical models, including ovarian cancer (136, 144).

### 5.2 Depleting TAMs: targeting the macrophages that aid tumour growth

**Figure 3:**
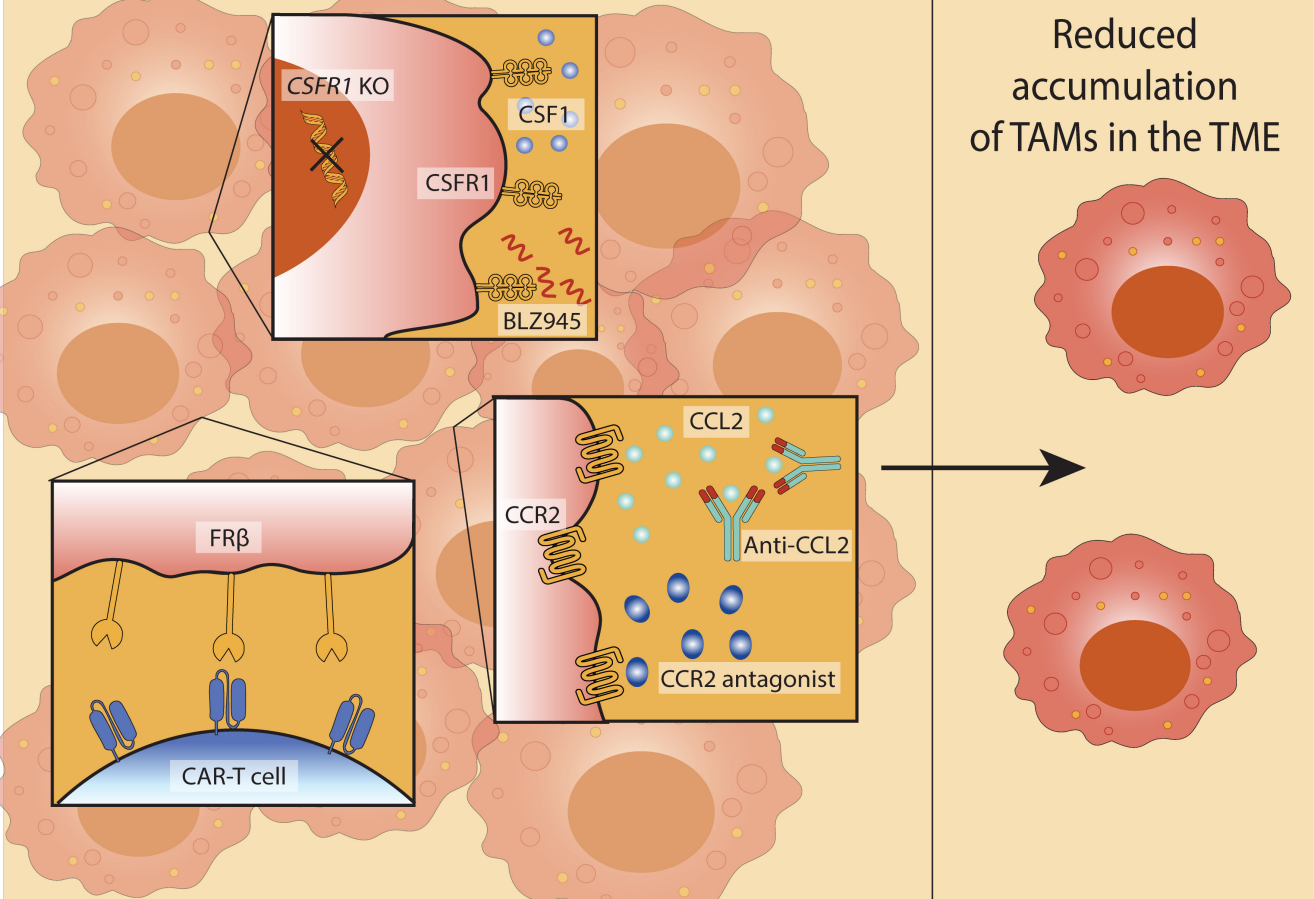
Therapeutic strategies to reduce TAM accumulation in the TME. Approaches to target TAM recruitment and survival to limit their accumulation in the TME include: inhibition of the colony-stimulating factor 1 receptor (CSF1R) pathway (through genetic knockout (CSF1R KO) or pharmacological inhibition using CSF1R inhibitor BLZ945) (top); disruption of the CCL2-CCR2 axis using anti-CCL2 antibodies or CCR2 antagonists (centre); chimeric antigen receptor (CAR)-T cells engineered to recognise the TAM marker folate receptor β (FRβ) (bottom). Collectively, these strategies contribute to reduced TAM accumulation in the TME, potentially enhancing anti-tumour immunity.

The frequency and distribution of TAMs strongly correlate with poor prognosis across multiple malignancies, including breast, prostate, ovarian and lung cancers (158–164). In preclinical breast cancer models, macrophage depletion significantly delays tumour progression, positioning TAMs as a promising therapeutic target (165). However, efforts to deplete macrophages (**Figure 3**), have revealed the need for refined approaches that distinguish between immunosuppressive and immunostimulatory subsets.

The colony-stimulating factor 1 (CSF1)-CSF1 receptor (CSF1R) axis represents a key regulatory pathway for macrophage survival and differentiation (166). Tumour cells, macrophages, and other stromal components secrete CSF1, sustaining TAM viability within the TME (167, 168). Genetic ablation of CSF1R in murine breast cancer models reduces TAM accumulation and promotes NK cell activation, with adoptive NK cell transfer further enhancing tumour control (145). Blockade of CSF1R using small-molecule inhibitors, such as BLZ945, has shown efficacy in glioma, where treatment led to TAMs with increased expression of antigen presentation genes (146) and downregulation of pro-tumour macrophage markers (147). Notably, these latter effects appear to reflect macrophage repolarisation rather than depletion.

Another strategy to limit TAM accumulation involves targeting monocyte recruitment through the CCL2–CCR2 chemokine axis. Tumour-derived CCL2 facilitates the recruitment of CCR2-expressing monocytes, which subsequently differentiate into TAMs. Inhibition of this pathway, via anti-CCL2 antibodies or CCR2 antagonists, reduces TAM numbers and restores NK cell effector function (150–152). In hepatocellular carcinoma models, CCL2 blockade enhances NK cell activation, IFN-γ production, and cytotoxicity, supporting the therapeutic value of this approach (150).

Nevertheless, blockade of monocyte recruitment can trigger compensatory mechanisms, such as increased neutrophil infiltration, which may counteract therapeutic benefits (62). One strategy to circumvent this is to selectively target TAM-associated receptors. For instance, chimeric antigen receptor (CAR)-T cells engineered to recognise folate receptor beta (FRβ), a marker enriched on immunosuppressive TAMs, successfully deplete this population, promote pro-inflammatory polarisation, and suppress tumour growth in preclinical models (154).

### 5.3 Modulation of TAM-NK cell crosstalk: shaping the immune communication to combat cancer

**Figure 4:**
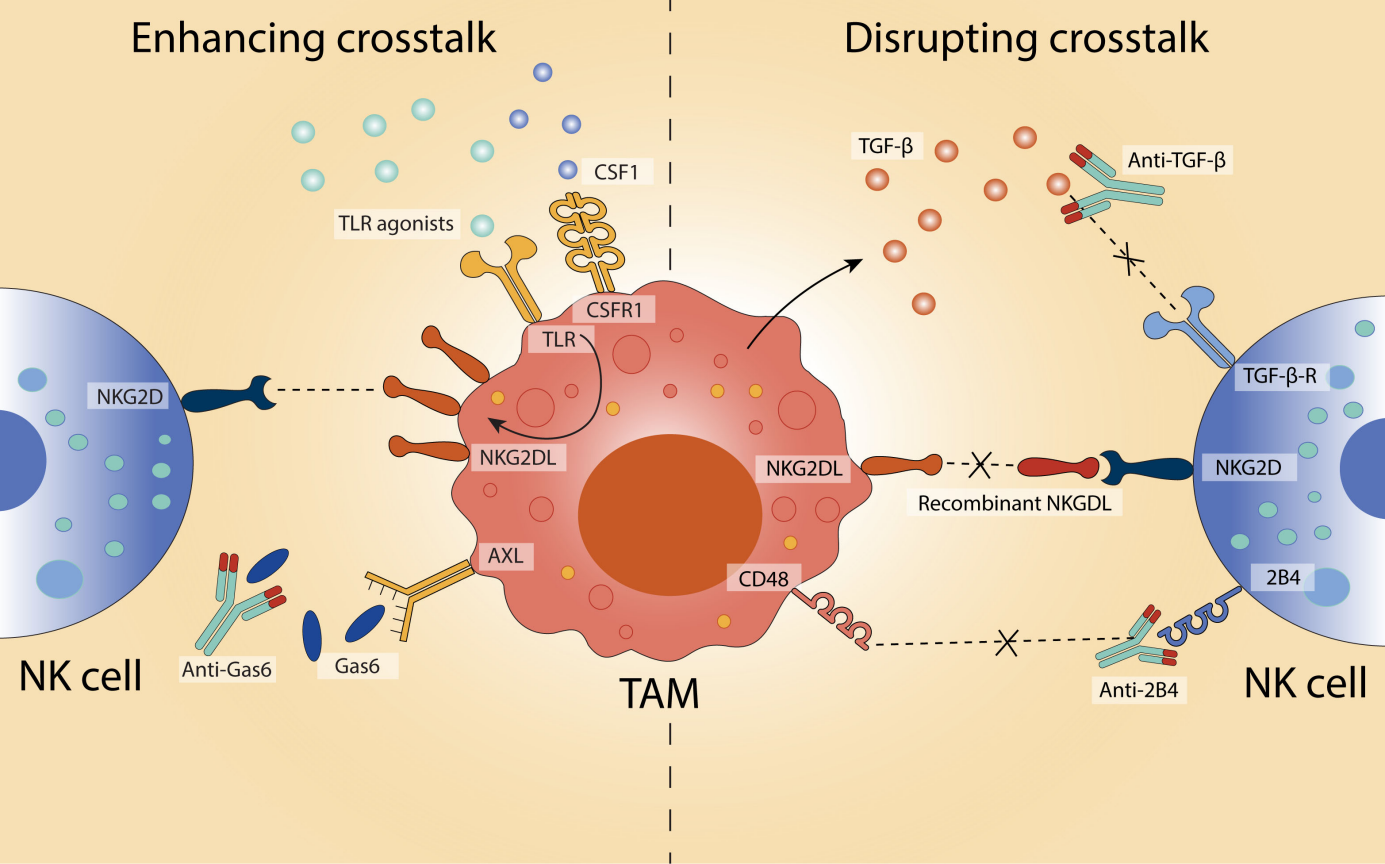
Modulating TAM–NK cell crosstalk to influence anti-tumour immunity. Strategies to either enhance or disrupt the interaction between TAMs and NK cells in the TME aim to restore or enhance NK cell cytotoxicity and contribute to anti-tumour responses. On the left, enhancing crosstalk is achieved through TLR agonists and CSFR1 stimulation, which upregulate NKG2D ligands (NKG2DL) on TAMs and promote NK cell activation. Inhibition of Gas6-AXL signalling using anti-Gas6 antibodies further supports immune activation. On the right, disruption of immunosuppressive crosstalk is demonstrated via blockade of TGF-β signalling (using anti-TGF-β antibodies) and interference with inhibitory CD48-2B4 interactions (via anti-2B4 antibodies). Additionally, recombinant NKG2DL can be used to inhibit NKG2D-mediated suppression.

Rather than indiscriminately depleting macrophages, a more refined therapeutic strategy involves selectively modulating the bidirectional interactions between TAMs and NK cells within the TME. By either amplifying beneficial communication or disrupting suppressive crosstalk, it may be possible to restore NK cell function while preserving the immune-regulatory roles of macrophages critical for tissue integrity (**Figure 4**).

One approach to enhance NK cell function is to increase expression of activating ligands on macrophages, particularly those that engage the NK cell receptor NKG2D. In mice, peritoneal macrophages stimulated with TLR agonists, including LPS (TLR4), Pam_3_CSK_4_ (TLR2), zymosan (TLR2/6), and poly(I:C) (TLR3), upregulate the NKG2D ligand RAE-1 (169). Tumour-derived CSF1 similarly drives RAE-1 expression on TAMs (109). Poly(I:C) treatment also induces the expression of other murine NKG2D ligands, such as H60 and MULT-1, while promoting secretion of type I interferons and cytokines critical for NK cell activation, including IFN-β, IL-12, IL-15, and IL-18 (48). Neutralisation of IFN-β or IL-15 diminishes NKG2D expression and NK cell cytotoxicity, demonstrating the centrality of this axis to immune activation (48). In human monocytes, analogous ligand upregulation can be achieved through IFN-α or TLR4 stimulation (170). LPS increases surface MICA and ULBP1–3 expression and enhances NK cell-derived IFN-γ in a MICA-NKG2D-dependent manner (170). However, prolonged or high-dose LPS exposure can paradoxically render macrophages more susceptible to NK cell-mediated lysis via NKG2D recognition, highlighting the delicate balance required in therapeutic modulation (47).

While the induction of activating ligands on macrophages can bolster NK cell function, persistent ligand expression can drive NK cell exhaustion. In both murine and human models, sustained exposure to RAE-1δ+ or MICA^high^ TAMs results in reduced NKG2D expression and diminished cytotoxic capacity (109, 171). In melanoma models, recombinant NKG2D ligands have been shown to partially restore NK cell responsiveness (109). Similar effects are observed with the CD48–2B4 axis: TAMs expressing CD48 transiently activate NK cells, but prolonged stimulation leads to functional exhaustion (114). In hepatocellular carcinoma, blockade of 2B4 reverses this dysfunction and restores NK cell IFN-γ production (114).

Disruption of suppressive cytokine signalling represents another avenue to restore NK cell cytotoxicity. TGF-β, a canonical immunosuppressive cytokine secreted by both tumour cells and TAMs, impairs NK cell function by downregulating activating receptors such as NKG2D and NKp30 (81). In human gastric cancer and murine breast cancer models, TGF-β blockade restores NK cell activity and augments anti-tumour responses (83, 145, 155). Targeting macrophage-intrinsic suppressive pathways is also showing promise. The receptor tyrosine kinase AXL, often upregulated on TAMs in breast, ovarian, renal, and lung cancers, correlates with poor clinical outcomes and skews macrophages toward an immunosuppressive phenotype (172). In leukaemia, AXL is induced by tumour-derived GAS6, which also acts on NK cells to reduce NKG2D expression and impair cytotoxicity (173, 174). Blockade of either AXL or GAS6 enhances NK cell activation, reduces metastasis, and promotes tumour control in breast cancer, melanoma, and pancreatic cancer models (156, 157).

Immune checkpoint inhibition remains a promising approach in cancer immunotherapy. While there is limited direct evidence of checkpoint molecule-mediated interactions between TAMs and NK cells, therapeutic strategies targeting these checkpoints often modulate the activity of both cell types, suggesting underlying immune crosstalk that could be targeted. For example, V-domain Ig suppressor of T cell activation (VISTA) is highly expressed on TAMs, and its blockade improves survival in murine models of leukaemia and lymphoma (175). Notably, although NK cells do not express VISTA, anti-VISTA antibodies have demonstrated increased NK cell maturation and activation (176, 177), implying that indirect modulation through other immune populations, possibly including macrophages, is at play.

Lymphocyte activation gene-3 (LAG-3), an inhibitory receptor expressed on NK cells, is associated with reduced IFN-γ production and proliferation (178). Blockade of LAG-3 has been effective in restoring NK cell function in chronic lymphocytic leukaemia, including increased production of IFN-γ and IL-12 (179). Although there is limited evidence for direct interaction between TAMs and NK cells via LAG-3, LAG-3^+^ T cells can bind MHC class II on macrophages (180), and soluble LAG-3 binding to MHC class II on macrophages inhibits monocyte-to-macrophage differentiation (181).

T-cell immunoglobulin and mucin-domain containing-3 (TIM-3) is expressed on both TAMs and NK cells (182, 183). *In vitro*, TIM-3 blockade enhances NK cell cytotoxicity against cancer cell lines and primary multiple myeloma cells, accompanied by increased IFN-γ production; *in vivo*, this corresponds to reduced tumour growth (184, 185). In macrophages, blocking TIM-3 inhibits polarisation toward an immunosuppressive phenotype in glioblastoma (186). Blockade of T cell immunoreceptor with Ig and ITIM domains (TIGIT), also present on both NK cells and TAMs, enhances NK cell cytotoxicity against melanoma cells *in vitro* and reduces metastatic growth in murine melanoma models (187). In TAMs, TIGIT supports an immunosuppressive phenotype (188), but inhibition can reprogramme these cells toward a pro-inflammatory, anti-tumour state (189).

### 5.4 Strengths, limitations, and translational challenges of harnessing macrophages

Several strategies to manipulate TAMs with the aim of enhancing NK cell function have emerged, including approaches to repolarise their phenotype, deplete suppressive subsets, and enhance TAM-NK cell crosstalk. Among these, antibody-based therapies targeting specific surface markers on pro-tumoural TAMs represent some of the most promising and clinically advanced avenues. Anti-MARCO antibodies have shown tumour-reducing effects across multiple models (136–138), although MARCO is also expressed on non-tumour macrophages (21), highlighting the need for tissue-specific profiling and validation. Anti-Clever-1 antibodies offer more selectivity by targeting immunosuppressive TAMs while sparing homeostatic populations (190), though their broader applicability across tumour types and their impact on NK cells remain underexplored. CAR-T strategies directed at TAM markers such as FRβ also offer a degree of specificity (154), yet their effects on NK cells, as well as their persistence, trafficking, and safety profiles, remain largely uncharacterised.

While several macrophage-targeted therapies, including anti-MARCO and anti-Clever-1 antibodies, have entered early-phase clinical trials, few include NK cell-specific endpoints. Most focus on T cell responses or cytokine outputs, neglecting NK-relevant metrics such as CD107a expression or intratumoural infiltration. For instance, clinical trials assessing CSF1R inhibition (e.g., NCT02526017) or checkpoint blockade (e.g., NCT02817633) do not evaluate NK functional markers (191, 192). Many of the strategies outlined in **Table 2** remain preclinical, with toxicity, tumour-type specificity, and durability yet to be addressed.

Compared to T cell-based approaches, TAM-targeted therapies offer distinct advantages. Their antigen-independent mechanism avoids issues of antigen loss or MHC downregulation (193, 194). TAM repolarisation allows *in situ* immune reprogramming, potentially overcoming the trafficking barriers faced by adoptively transferred T cells (195). Repolarised TAMs can also secrete IL-15 and IL-12, enhancing NK cytotoxicity without the systemic toxicity of exogenous cytokines (41). Furthermore, because activation is localised, repolarisation carries a lower risk of cytokine release syndrome compared to CAR-T therapies (196).

However, macrophage plasticity poses a significant barrier to durable responses; macrophages are highly responsive to local cues, including the immunosuppressive signals of the TME, and therefore may not maintain an anti-tumour phenotype over time (197–199). Additionally, unlike memory-forming T cells, macrophages do not clonally expand (1, 3). Repolarising agents like TLR agonists [e.g., LPS, poly(I:C)] can upregulate NK-activating ligands but often lack tumour specificity, leading to off-target inflammation (200–203). Even more tolerable agents like β-glucan or inhibitors of broadly expressed molecules (e.g., Axl, TGF-β) may cause systemic toxicity without targeted delivery strategies (204–206).

TAM depletion strategies offer a different approach, aiming to remove suppressive macrophages and reduce inhibitory signalling toward NK cells. However, these are often not selective enough, for example CSF1R blockade can deplete supportive myeloid cells that produce IL-15 and IL-18, cytokines that are important for NK survival (207). In lung cancer models, CSF1R inhibitor BLZ945 impaired NK infiltration and increased metastasis (207). Depleting CD206^+^ TAMs similarly disrupted NK recruitment, indicating that not all TAMs are suppressive (208). Thus, complete depletion may inadvertently remove macrophages that support NK-mediated immunity.

Moreover, TAM populations may exhibit resistance to CSF1R inhibition (209) or are replenished via alternative recruitment (e.g.,CCR5) (210), while compensatory upregulation of PD-L1 or other inhibitory molecules may undermine efficacy (167). Although depletion strategies bypass the need for antigen specificity, they carry a higher risk of disrupting tissue-resident macrophages involved in homeostasis (208). The functional heterogeneity of TAMs demands greater precision in distinguishing suppressive subsets from those with beneficial roles (211).

Enhancing TAM–NK cell crosstalk offers a mechanistically attractive alternative. Instead of depleting or repolarising macrophages, this strategy modulates communication pathways to restore NK cell activity. This approach may be especially effective in NK-sensitive tumours where T cell responses are limited (212). However, it requires the presence of functional NK cells, limiting efficacy in tumours with low infiltration unless paired with adoptive NK cell transfer (213, 214).

Each TAM-targeting approach has trade-offs. Repolarisation preserves beneficial functions, but is vulnerable to reversal due to plasticity. Depletion removes immunosuppressive signalling, but risks harming supportive macrophages. Crosstalk enhancement provides targeted immune recalibration with minimal disruption, but depends on the presence of responsive NK cells. Of these, crosstalk modulation may strike the most effective balance: restoring NK function while maintaining macrophage-mediated tissue integrity. Ultimately, advancing TAM-targeted therapies will require a more nuanced understanding of macrophage-NK cell dynamics. Emerging technologies such as single-cell RNA sequencing, spatial transcriptomics, and proteomics offer powerful tools to explore this complexity.

## 6 Resolving the spatiotemporal crosstalk between macrophages and NK cells through single-cell and spatial multi-omics

While macrophage and NK cell interactions have been studied using flow cytometry, bulk RNA-sequencing, and co-culture models, these methods fall short in capturing spatial context, heterogeneity, and post-translational modifications. Emerging high-dimensional approaches such as spatial transcriptomics (ST), single-cell RNA sequencing (scRNA-seq), and proteomics, promise to overcome these limitations, offering a systems-level perspective on immune cell interplay.

### 6.1 Single-cell sequencing: unravelling cellular heterogeneity

Single-cell RNA sequencing (scRNA-seq) has transformed our understanding of immune cell heterogeneity and function by enabling transcriptomic profiling at single-cell resolution. This technology is particularly valuable for dissecting the diverse phenotypic states of macrophages within the tumour microenvironment (TME), a complexity underscored by findings such as the enhanced ability of spleen macrophages, compared to their lung counterparts, to potentiate NK cell cytotoxicity (215). For instance, scRNA-seq identified a neuron-like TAM subset in lung adenocarcinoma that promotes tumoural neurogenesis (216). Similarly, scRNA-seq has provided insights into the regulation of tumour-infiltrating NK cells, revealing that inhibition of the transcription factor HIF-1α can enhance cytotoxicity, suggesting novel therapeutic avenues (217). Integrative scRNA-seq analyses on NK cells from over 700 patients with 24 types of cancer shows heterogeneity in NK cell composition in a tumour-type-specific manner and importantly, also identified a population of tumour-associated NK cells that show impaired anti-tumour functions (218).

Beyond cellular profiling, integrating ligand-receptor interaction frameworks such as CellPhoneDB (219) and NicheNet (220) with scRNA-seq data has enabled the prediction of intercellular communication networks. In ovarian carcinoma, this approach revealed robust crosstalk, mediated by CXCL and CCL chemokines, between anti-tumour TAM subsets and cytotoxic NK cells (221). Moving forward, combining scRNA-seq with computational inference of cell-cell interactions holds significant promise for uncovering regulatory mechanisms such as cytokine feedback loops, immune checkpoint modulation, and metabolic coordination within the TME.

### 6.2 Spatial transcriptomics: adding the context of location

While scRNA-seq provides powerful insights into cellular heterogeneity, it lacks spatial resolution. Spatial transcriptomics (ST) addresses this limitation by mapping gene expression directly onto intact tissue sections, preserving the native architecture and cellular context. This spatial dimension is particularly crucial for elucidating cell–cell interactions within complex environments such as the TME.

ST has proven instrumental in characterising macrophage infiltration patterns in non-small cell lung cancer (NSCLC). In patient samples from anti-PD-1/PD-L1 immunotherapy trials, spatial profiling revealed the distribution of CD163^+^ macrophages across tumour and stromal areas, with high densities correlating with poor clinical outcomes (222). These findings underscore the multifaceted roles of TAMs in modulating immune responses and contributing to therapeutic resistance. In pancreatic adenocarcinoma, integration of scRNA-seq with ST enabled the identification of an anti-tumour macrophage population marked by IRF7 activity, which limited tumour progression through lipid metabolism-dependent mechanisms (223). More broadly, multi-modal spatial analyses have revealed that TAMs are not homogeneously distributed across the TME. Rather, distinct TAM subsets occupy defined niches: pro-inflammatory TAMs are enriched in tumour cores, whereas anti-inflammatory TAMs preferentially localise to invasive margins in gastric and pancreatic cancers (224, 225). Moreover, TAM function appears spatially encoded - those residing in perivascular or hypoxic regions exhibit immunosuppressive phenotypes, while macrophages at the invasive front can display anti-tumour activity (225). These spatially resolved phenotypes suggest that local tissue architecture and microenvironmental cues shape macrophage polarisation and function.

**Figure 5:**
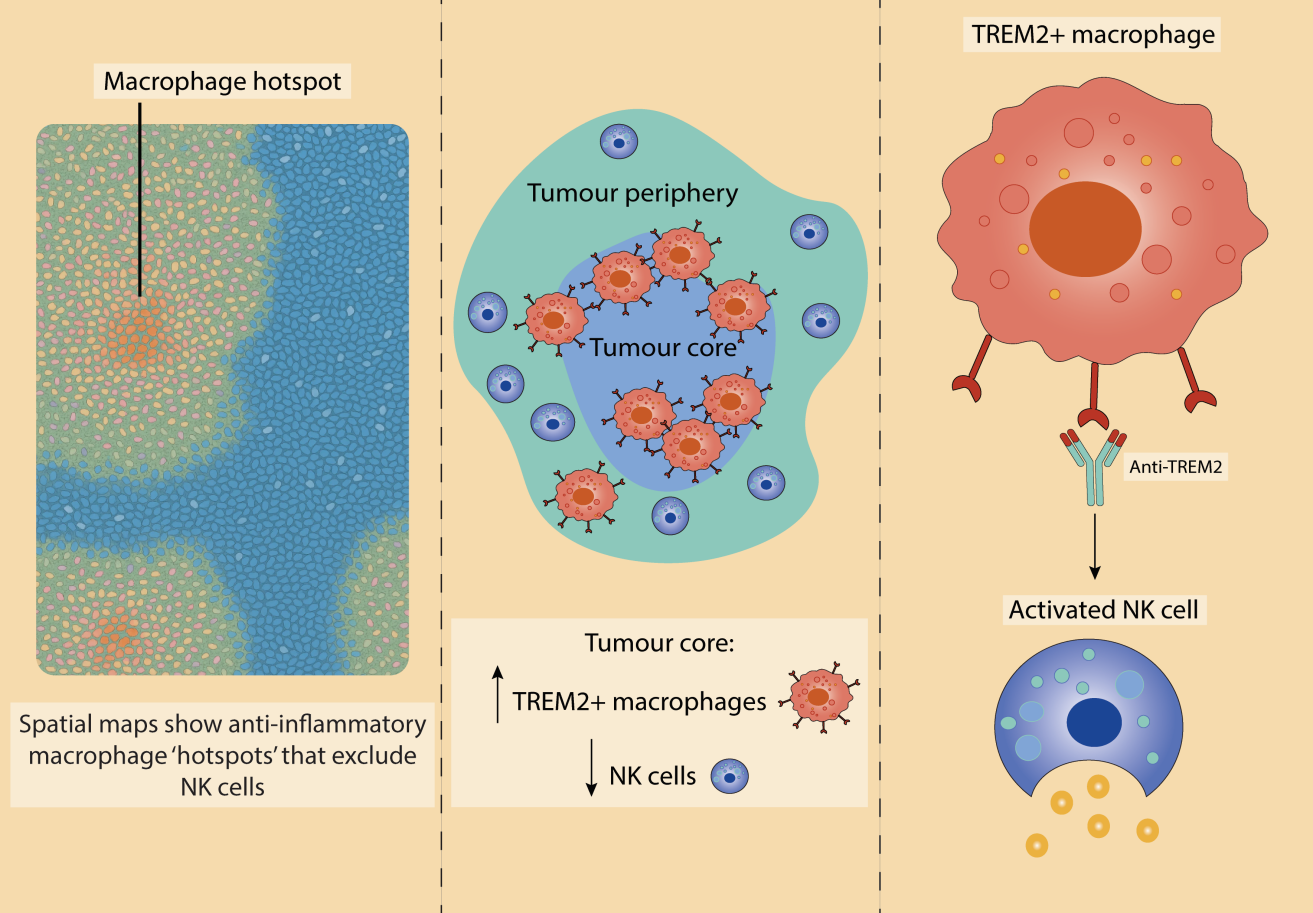
Spatial transcriptomic insights into TAM-NK cell crosstalk in the TME. Recent spatial transcriptomic analyses reveal altered distributions of TAMs and NK cells across tumour regions. Left: Anti-inflammatory macrophage-rich niches predominate in the TME of both adenocarcinoma and squamous cell carcinoma, with a corresponding reduction in NK cell infiltration and cytotoxic gene expression. Based on work by De Zuani et al. (226). Middle: In non-small cell lung cancer, TREM2^+^ TAMs are enriched within the tumour core, acting as a barrier to NK cell infiltration and promoting an immunosuppressive landscape. Right: Therapeutic blockade of TREM2 reverses this exclusion, enhancing NK cell activation. Middle and right panels based on findings from Park et al. (227).

Importantly, recent spatial transcriptomic studies have begun to map TAM and NK cell crosstalk within the TME, revealing key mechanisms of immune exclusion and dysfunction (**Figure 5**). In NSCLC, TREM2^+^ macrophages are highly enriched in tumour cores, where they physically and functionally restrict NK cell infiltration; antibody-mediated TREM2 blockade reactivates NK cells, highlighting a targetable axis of suppression (227). Similarly, in adenocarcinoma and squamous-cell carcinoma, clusters of anti-inflammatory macrophages form immunosuppressive hotspots, inversely correlating with NK cell abundance (226). These spatially resolved TAM-NK interactions further support the notion that immune spatial context may influence response to immunotherapy.

Future studies examining macrophage–NK cell crosstalk will benefit from ST’s ability to reveal sites of cellular co-localisation, spatially restricted cytokine gradients, and the organisation of functional niches. Combining ST with scRNA-seq facilitates high-resolution mapping of cellular diversity within the TME. One notable advancement is Zman-seq, a dynamic single-cell technology that captures transcriptomic changes over time (228). In glioblastoma, Zman-seq uncovered a rapid sequence of immunological events: NK cells acquired a dysfunctional phenotype within 24 hours, driven by TGFβ1 signalling, followed by the differentiation of infiltrating monocytes into immunosuppressive TAMs within 36–48 hours, characterised by upregulation of suppressive myeloid checkpoints (228). These findings point to a critical early window for therapeutic intervention to preserve immune cell function.

Further integration of ST with high-dimensional imaging techniques, such as co-detection by indexing (CODEX) (229) or multiplexed ion beam imaging (MIBI) (230), will allow transcriptomic data to be overlaid with protein-level information. This multimodal approach will enhance validation of spatial signatures and enable a more comprehensive characterisation of immune cell states and interactions within the TME. However, current ST platforms still face major limitations. Researchers must often choose between single-cell resolution and full transcriptome coverage, meaning high resolution and comprehensive data is hard to achieve at the same time. Even at single-cell resolution, the number of genes reliably captured per cell is often too low to support detailed analyses, especially when compared to liquid-based single-cell RNA-seq (231). In addition, important parts of the TME, such as the extracellular matrix, are still not well captured by most current technologies, leaving major gaps in how we understand tumour structure and cell communication.

### 6.3 Proteomics: capturing functional states and post-translational dynamics

In the context of tumours, it is proteins, not RNA, that ultimately drive cell behaviour. While transcriptomic data offers important insights, gene expression levels often do not correlate with protein abundance or activity. Proteins interact directly, undergo post-translation modifications, and include secreted factors that mediate communication between cells – critical factors that are missed by RNA-based methods. This underscores the importance of integrating proteomic data to gain a more accurate and comprehensive understanding of cellular function and tumour biology.

Proteomic analyses have shed light on the heterogeneity and function of macrophage subsets. In models of liver fibrosis, distinct populations, including embryo-derived liver-resident Kupffer cells (EmKCs) and monocyte-derived Kupffer cells (MoKCs), were delineated based on unique proteomic signatures (232). In melanoma, proteomic profiling of TAMs revealed a shift toward enhanced cholesterol metabolism and reduced immune activation during tumour progression (233). Similar approaches have elucidated diversity within the NK cell compartment. For example, proteomics has distinguished memory-like NK cells from naïve populations by differential expression of key regulatory proteins (234). Furthermore, NK cell-derived extracellular vesicles, characterised through proteomic profiling, have been shown to contain effector molecules such as Fas ligand, TRAIL, NKG2D, and β-actin, which collectively contribute to their anti-tumour function (235).

Looking ahead, mass spectrometry-based proteomics and targeted platforms such as cytometry by time-of-flight (CyTOF) will be instrumental in dissecting macrophage-NK cell interactions. These approaches enable the detection of activation markers, secreted cytokines, and metabolic enzymes, providing functional insights beyond transcriptomic profiling. When integrated with scRNA-seq and ST, proteomics enables a multidimensional view of immune cell states and interactions - essential for understanding the dynamic regulation of immunity within the TME and informing next-generation immunotherapies.

While scRNA-seq, ST, and proteomics each provide distinct insights into macrophage and NK cell biology, combining these three technologies will allow macrophage-NK cell crosstalk to be studied at a cellular, spatial and functional level. For instance, a study in gastric cancer using integrated spatial multi-omics has shown that the TME has distinct, tissue-specific metabolism signatures (236). In glioblastoma, integrative spatial analysis showed cell organisation is associated with hypoxic cancer cells, with a distinct macrophage state marked by pro-inflammatory cytokine expression being identified (237). Combining scRNA-seq, ST, and proteomics provides a layered view of the TME, allowing transcriptional states, spatial relationships, and protein activity to be mapped together. This approach is well-suited to dissecting macrophage-NK cell interactions, revealing functional states and immunoregulatory niches that shape their crosstalk.

## 7 Concluding remarks

Harnessing the interplay between macrophages and NK cells within the TME represents a promising strategy to heighten innate immunity and drive durable anti-tumour responses. Unlike conventional T cell–focused therapies, targeting the mechanisms that govern macrophage-NK cell communication has the potential to initiate more coordinated, tissue-integrated immune activation capable of overcoming the immunosuppressive barriers within the TME. TAMs are abundant and actively shape the immune landscape, making them more accessible and impactful therapeutic targets compared to T cells, which often face challenges such as exhaustion, antigen escape, and poor infiltration.

However, current TAM-targeting strategies have limitations. Approaches aimed at repolarising macrophages toward a simplified “M1-like” phenotype underestimate the complexity and plasticity of TAMs, whose states rapidly shift in response to local cues, reducing the durability of such interventions. Depletion strategies lack selectivity, risking the loss of macrophage subsets essential for supporting NK cell function and maintaining tissue homeostasis. Given these challenges, the most promising path forward lies in directly modulating TAM-NK cell crosstalk through manipulating cell communication to restore NK cell cytotoxicity and promote anti-tumour activity. So far this has been achieved through upregulating NKG2D ligands on macrophages or blocking suppressive TGF-β secretion.

However, to fully exploit this therapeutic potential, future research must address several key areas. Mapping the spatial and temporal dynamics of macrophage-NK cell interactions using advanced technologies such as spatial transcriptomics and proteomics will provide detailed insights into their behaviour within the TME. It is also essential to identify selective strategies that target immunosuppressive TAM subsets, while preserving macrophage populations that support NK cell function. Understanding the mechanisms underlying NK cell dysfunction driven by chronic TAM engagement will guide the development of approaches to restore NK cytotoxic capacity. Furthermore, establishing physiologically relevant model systems, including tumour organoids and patient-derived co-cultures, will better enable preclinical testing of combination therapies. Finally, integrating TAM-NK cell-targeting strategies with existing immuno-oncology approaches, such as checkpoint blockade and adoptive cell therapies, may amplify therapeutic outcomes and broaden patient responsiveness. Collectively, these directions will deepen our understanding of innate immune crosstalk in cancer and provide a foundation for the development of next-generation immunotherapies.
